# The combined role of PET/CT metabolic parameters and inflammatory markers in detecting extensive disease in small cell lung cancer

**DOI:** 10.3389/fonc.2022.960536

**Published:** 2022-09-14

**Authors:** Yao Hu, Jin Sun, Danming Li, Yangyang Li, Tiannv Li, Yuxiao Hu

**Affiliations:** ^1^ Department of PET/CT Center, Jiangsu Cancer Hospital and Jiangsu Institute of Cancer Research and the Affiliated Cancer Hospital of Nanjing Medical University, Nanjing, China; ^2^ Department of Nuclear Medicine, The First Affiliated Hospital of Nanjing Medical University, Nanjing, China; ^3^ Department of Radiation Oncology, The First Affiliated Hospital of Nanjing Medical University, Nanjing, China

**Keywords:** SCLC, inflammatory markers, metabolic parameters, PET/CT, MTV/MLR

## Abstract

The combined role of inflammatory markers [including neutrophil/lymphocyte ratio (NLR), platelet/lymphocyte ratio (PLR), monocyte/lymphocyte ratio (MLR), and systemic immune-inflammation index (SII)] and PET/CT metabolic parameters [including maximum standardized uptake value (SUVmax), mean standardized uptake value (SUVmean), metabolic tumor volume (MTV), and TLG (total lesion glycolysis)] at baseline in evaluating the binary stage [extensive-stage disease (ED) and limited-stage disease (LD)] of small cell lung cancer (SCLC) is unclear. In this study, we verified that high metabolic parameters and inflammatory markers were related to the binary stage of SCLC patients, respectively (*p* < 0.05). High inflammatory markers were also associated with high MTV and TLG in patients with SCLC (*p* < 0.005). Moreover, the incidences of co-high metabolic parameters and inflammatory markers were higher in ED-SCLC (*p* < 0.05) than those in LD-SCLC. Univariate logistic regression analysis demonstrated that ^Co-high^ MTV/NLR, ^Co-high^ MTV/MLR, ^Co-high^ MTV/SII, ^Co-high^ TLG/NLR, ^Co-high^ TLG/MLR, and ^Co-high^ TLG/SII were significantly related to the binary stage of SCLC patients (*p* = 0.00). However, only ^Co-high^ MTV/MLR was identified as an independent predictor for ED-SCLC (odds ratio: 8.67, 95% confidence interval CI: 3.51–21.42, *p* = 0.000). Our results suggest that co-high metabolic parameters and inflammatory markers could be of help for predicting ED-SCLC at baseline. Together, these preliminary findings may provide new ideas for more accurate staging of SCLC.

## Introduction

Lung cancer is one of the main causes of cancer-related death in the world (1, 2). According to pathology, lung cancer is mainly divided into adenocarcinoma, squamous cell carcinoma, small cell lung cancer (SCLC), and so on. Among them, SCLC accounts for about 15%, with the characteristics of early metastasis, easy recurrence, and low 5-year survival rate (as low as 5%–10%) (2). According to a binary stage method in most of the articles, SCLC is classified into limited-stage disease (LD-SCLC) confined to the ipsilateral hemithorax and extensive-stage disease (ED-SCLC) spread beyond the ipsilateral hemithorax, the former including contralateral mediastinal and ipsilateral supraclavicular lymph nodal metastases and the latter including hematogenous metastases and malignant pleural or pericardial effusion (3). The management of different stages are completely different in SCLC patients (4). Chemoradiotherapy is the standard treatment of LD-SCLC patients. While a proposed treatment program for ED-SCLC is systemic chemotherapy, which could offer rapid responses and the best palliation. The median survival times of LD-SCLC and ED-SCLC are only 15–20 months and 8–13 months (4), respectively. Therefore, correct staging is pivotal regarding the selection of appropriate and effective treatment strategies for individual patients with SCLC.

^18^F-fluorodeoxyglucose (^18^F-FDG) positron-emission tomography (PET)/computed tomography (CT), providing both functional and morphological data, is a systemic non-invasive imaging technique and used in tumor staging, treatment responses and recurrence diagnosis (5, 6). Maximum standardized uptake value (SUVmax), mean standardized uptake value (SUVmean), metabolic tumor volume (MTV), and total lesion glycolysis (TLG) are used as semi-quantitative parameters of PET/CT, which reflect the local metabolism and the biological aggressiveness of tumors (7). However, the use of PET/CT is still controversial since some previous studies showed that false-positive results affected stage for SCLC patients by using PET/CT (5). Hence, the metabolic parameters *via* PET/CT are not sufficient to evaluate the binary stage of SCLC. The FDG uptake in lesions is affected by many different factors including infection and inflammation (8). The SCLC patients with normal blood counts are advised to conduct a bone marrow biopsy in order to exclude bone marrow involvement (4). Recent studies have confirmed that inflammation plays significant roles in tumor microenvironment, where it influences tumor development, progression, and treatment response (9). Meanwhile, a growing body of evidence indicated that increased levels of serum inflammatory markers such as neutrophil/lymphocyte ratio (NLR), platelet/lymphocyte ratio (PLR), monocyte/lymphocyte ratio (MLR), and systemic immune-inflammation index (SII) correlated with the stage of malignancies [e.g., non-small cell lung cancer (NSCLC) (10), renal cell carcinoma (11), and colon cancer (12)]. Drawing on the above discoveries, the combined evaluation of metabolic parameters and inflammatory markers may be highly effective in detecting binary stage for SCLC at baseline.

However, to our knowledge, there are rare studies on the correlation between PET/CT semi-quantitative parameters and inflammatory markers in detecting the binary stage of SCLC. Therefore, the purpose of this study was to evaluate the relationship between inflammatory markers (NLR, PLR, MLR, and SII) in peripheral blood and semi-quantitative parameters (SUVmax, SUVmean, MTV, and TLG) *via* PET/CT and their combined role on detecting ED-SCLC.

## Materials and methods

### Subjects

All patients with SCLC underwent PET/CT scanning between January 2016 and June 2019 at the First Affiliated Hospital of Nanjing Medical University. Clinical data such as gender, age, smoking history, and hematological parameters [e.g., neutrophil (N), monocyte (M), lymphocyte (L), and platelet (P) counts] closest to the day of PET/CT scanning were collected. The Department of Clinical Laboratory of the First Affiliated Hospital of Nanjing Medical University performed the data analyses. Inflammatory markers based on N, M, L, and P—NLR, MLR, PLR, and SII—were calculated using the formula N/L, M/L, P/L, and P×N/L, respectively. The inclusion criteria were as follows (1): diagnosed with SCLC by surgical or biopsy specimens (2); did not undergo any treatment before PET/CT scanning and inflammatory marker measurement (3); PET/CT scanning performed within 1 week after inflammatory marker measurement (4); diagnosis of pleural effusion, pericardial effusion, lymph node, and distant organ metastasis by pathological examination and imaging examination such as contrast enhanced CT (CECT), PET/CT, and magnetic resonance imaging (MRI); and (5) without other tumors and without other diseases that alter hematological parameters. The study was approved by the Ethics Committee of the First Affiliated Hospital of Nanjing Medical University.

### ^18^F-FDG PET/CT scanning

^18^F-FDG PET/CT (Biograph 16HR; Siemens, Germany) examinations were acquired after fasting for 6 h and 60–75 min after intravenous injection of ^18^F-FDG (3.70–5.55 MBq/kg weight). The blood glucose level was below 120 mg/dl in all included patients before tracer injection. All patients had normal tidal breathing during PET and CT scans. Patients underwent low-dose CT scans (120–140 kV, 65 mA and 5.0 mm slice), followed by PET scans with six to eight bed positions (2 min per bed positions) per patient based on the height. The PET images were reconstructed with attenuation corrected CT using the ordered subset expectation maximization (OSEM) algorithm. Then, all dates were transferred in DICOM format to the Beth Israel PET/CT viewer plugin for FIJI and displayed as axial, coronal, and sagittal images. The SUVmax, SUVmean, MTV, and TLG of all lesions were delineated semiautomatically by the Beth Israel PET/CT viewer plugin for FIJI (http://sourceforge.net/projects/bifijiplugins/) (ImageJ distribution) (13).

### Statistical analysis

Data were analyzed using SPSS 25.0 software (SPSS, IL, USA). Data in accordance with normal distribution were expressed as the mean ± standard deviation (SD) values, while non-normal distribution data were expressed as median (inter-quartile interval). Statistical differences between groups were assessed by *t*-test or Mann–Whitney *U* test. *t*-test was performed for data in accordance with normal distribution, while non-normal distribution data were analyzed by Mann–Whitney *U* test. A Chi-square test was performed for rate comparisons. Receiver operating characteristic (ROC) curve analysis was performed to find optimal cutoff values of NLR, MLR, SII, MTV, and TLG to predict ED-SCLC. The area under curve (AUC) was calculated as a measure of the accuracy of the test. Logistic regression analysis was used to assay the association of patients’ clinical features, inflammatory markers, and metabolic parameters in detecting ED-SCLC. *p*-value < 0.05 was considered to be statistically significant.

## Results

### Patients’ characteristics

A total of 119 patients met the inclusion criteria and were enrolled in our study. Of these 119 patients, the median age was 64 (range: 25–91) years; 105 (64.6%) were male; 14 were female; 92 had a history of smoking; 47 SCLC patients were diagnosed with ED-SCLC, which spread beyond the ipsilateral hemithorax (**Table 1**). Median SUVmax, SUVmean, MTV, and TLG values for all lesions and median NLR, PLR, MLR, and SII values of the patients are shown in **Table 1**.

**Table 1:** Patient characteristics.

	Number (*n* = 119)	Value
**Gender**		
Male	105 (88.2%)	
Female	14 (11.8%)	
**Age**		
≤64	59 (49.6%)	
>64	60 (50.4%)	
**Smoking**		
Yes	92 (77.3%)	
No	27 (22.7%)	
**Tumor Stage**		
LD-SCLC	72 (60.5%)	
ED-SCLC	47 (39.5%)	
**Inflammatory markers**		
NLR		2.63 (0.30, 17.86)
PLR		132.49 (48.70, 561.19)
MLR		0.28 (0.04, 2.49)
SII		541.01 (47.88, 2,410.20)
**Metabolic parameters**		
SUVmax		12.78 (5.39, 47.34)
SUVmean		6.87 (3.17, 21.49)
MTV		65.58 (2.95, 1,208.91)
TLG		468.25 (19.77, 6,965.86)

#### Correlation of inflammatory markers with clinical features and binary stage of SCLC

**Figure 1:**
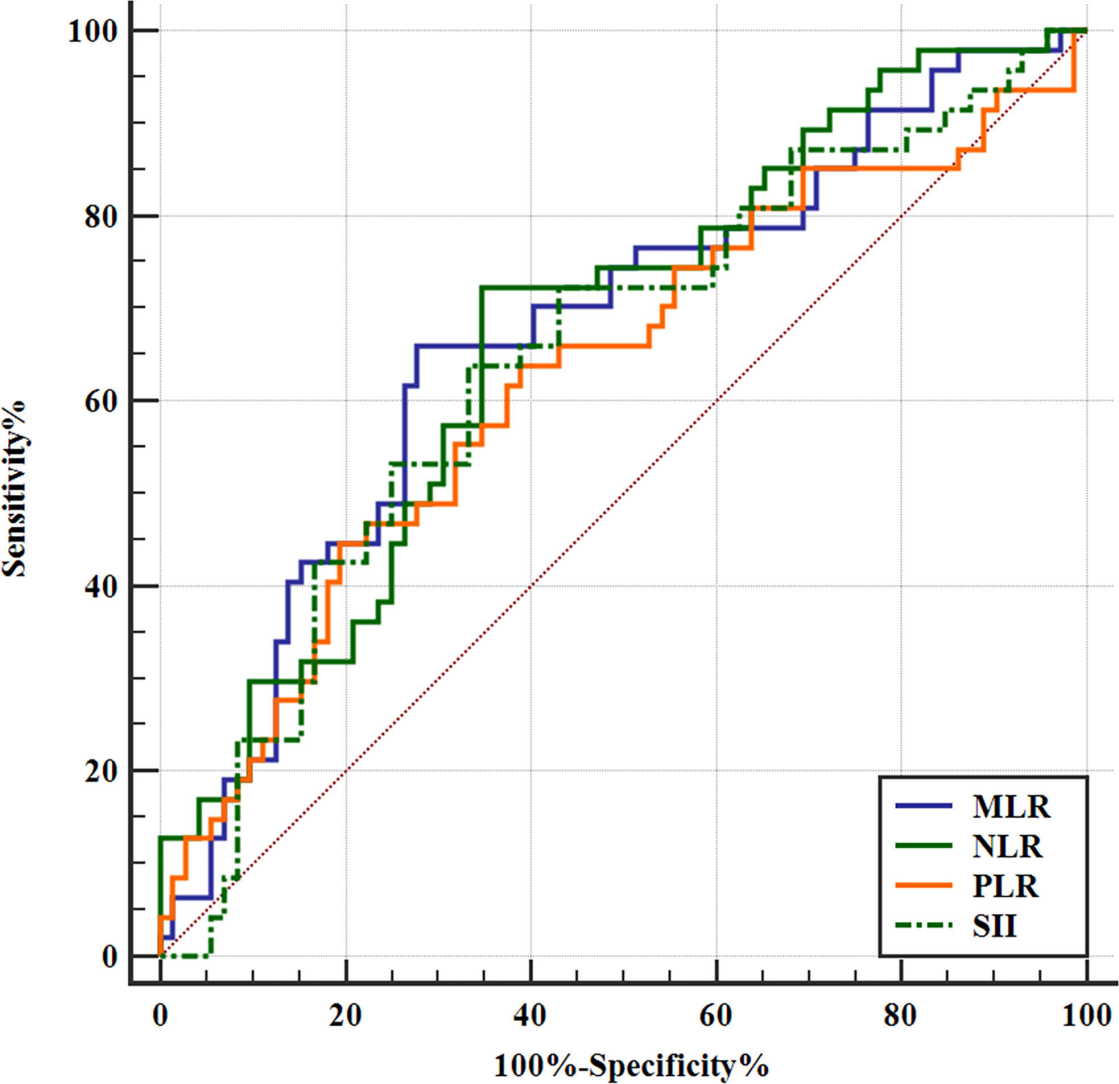
Receiver operating characteristic (ROC) curves of inflammatory markers for predicting binary stage of SCLC. NLR, PLR, MLR, and SII could predict the binary stage of SCLC. The ROC curve analysis of the NLR to predict ED-SCLC. With an NLR of 2.64 as the threshold, the sensitivity and specificity in the prediction of ED-SCLC were 72.34% and 65.28%, respectively. The AUC was 0.672 (95% confidence interval [CI]: 0.580–0.756; *p* = 0.0006). The ROC curve analysis of the PLR to predict ED-SCLC. With an PLR of 170.67 as the threshold, the sensitivity and specificity for the prediction of ED-SCLC were 44.68% and 80.56%, respectively. The AUC was 0.628 (95% CI: 0.535–0.715; *p* = 0.0178). The ROC curve analysis of the MLR to predict ED-SCLC. With an MLR of 0.31 as the threshold, the sensitivity and specificity for the prediction of ED-SCLC were 65.96% and 70.83%, respectively. The AUC was 0.669 (95% CI: 0.577–0.753; *p* = 0.0010). The ROC curve analysis of the SII to predict ED-SCLC. With an SII of 583.1 as the threshold, the sensitivity and specificity for the prediction of ED-SCLC were 63.83% and 66.67%, respectively. The AUC was 0.646 (95% CI: 0.553–0.731; *p* = 0.0055).

In patients with SCLC, NLR, PLR, MLR, and SII were significantly higher in ED-SCLC than LD-SCLC (*p* > 0.05, **Table 2**). Furthermore, we found that MLR is higher in patients older than 64 years. NLR, MLR, and SII are higher in male patients than female patients. NLR and SII are higher in patients with smoking than those without smoking (**Table 2**). With a cutoff value of 2.64, 170.67, 0.31, and 583.1, high NLR, PLR, MLR, and SII could respectively predict ED-SCLC (*p* < 0.05, **Figure 1**).

**Table 2:** Analysis of inflammatory markers in patients with SCLC (*n* = 119).

	NLR	*p*	PLR	*p*	MLR	*p*	SII	*p*
**Age**		0.992		0.103		0.019		0.116
≤64	2.48 (1.00, 8.69)		143.59 (54.40, 390.00)		0.26 (0.04, 2.06)		580.32 (136.54, 2410.20)	
>64	2.87 (0.30, 17.86)		117.91 (48.70, 561.19)		0.35 (0.08, 2.49)		496.51 (47.88, 2201.47)	
**Gender**		0.005		0.062		0.007		0.007
Male	2.86 (2.01, 3.61)		132.71 (105.73, 168.22)		0.28 (0.22, 0.42)		565.83 (403.96, 802.21)	
Female	1.72 (0.66, 14.68)		99.35 (48.70, 441.18)		0.21 (0.04, 0.47)		316.10 (123.22, 2201.47)	
**Smoking**		0.036		0.125		0.079		0.025
Yes	2.78 (0.30, 16.40)		138.88 (54.40, 561.19)		0.29 (0.08, 2.49)		595.21 (47.88, 2410.20)	
No	1.96 (0.66,17.86)		113.66 (48.70,366.97)		0.25 (0.04, 1.86)		425.75 (123.22, 1988.99)	
**Tumor Stage**		0.001		0.019		0.002		0.007
LD-SCLC	2.23 (0.30, 6.87)		123.11 (48.70, 390.00)		0.26 (0.04, 2.06)		460.84 (47.88, 2410.20)	
ED-SCLC	3.17 (1.00, 17.86)		149.33 (54.40, 561.19)		0.37 (0.08, 2.49)		737.47 (136.54, 2201.47)	

#### Correlation of metabolic parameters of SCLC with clinical features and binary stage of SCLC

**Figure 2:**
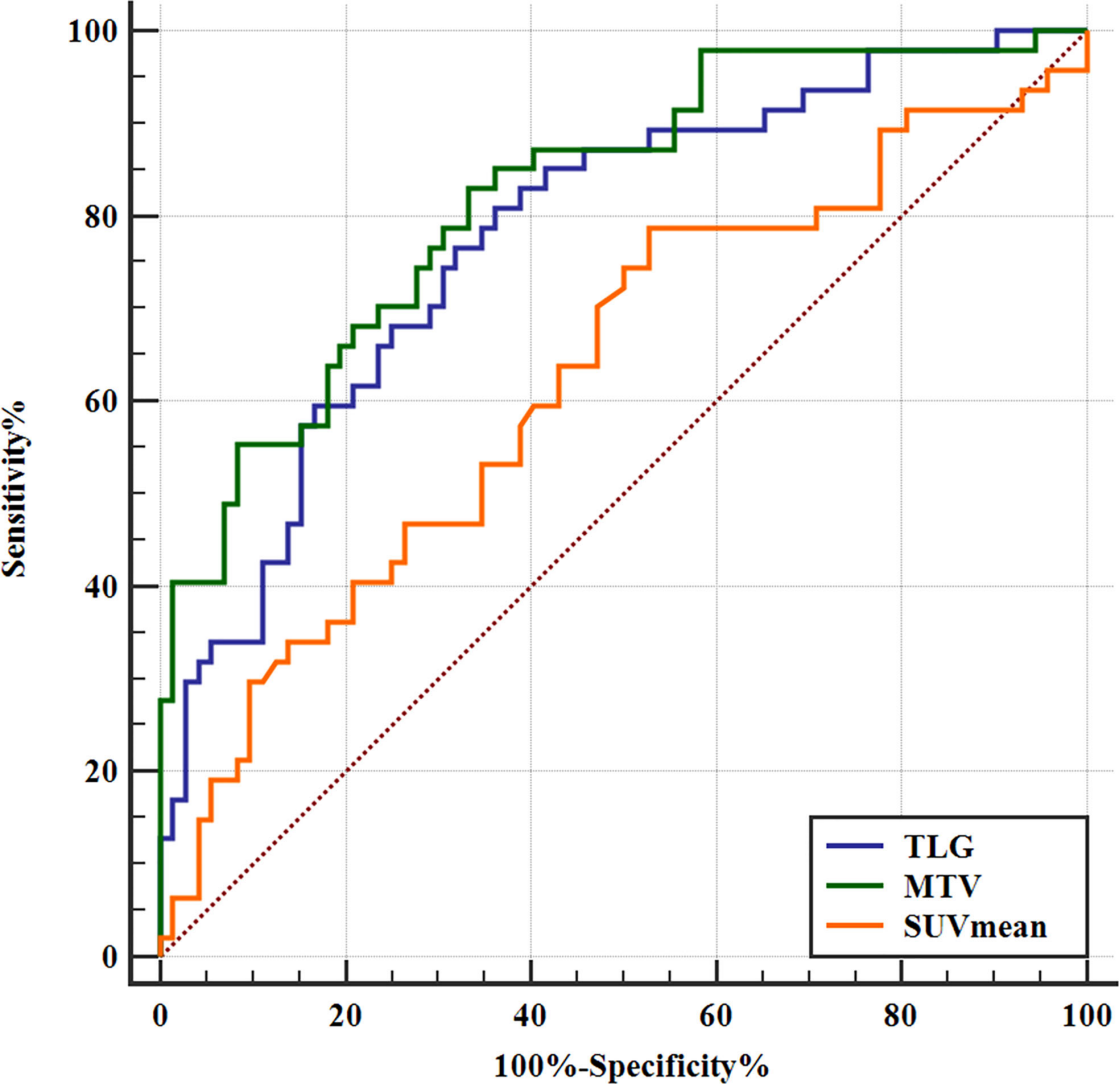
Receiver operating characteristic (ROC) curves of SUVmean, MTV, and TLG for predicting binary stage of SCLC. The SUVmean, MTV, and TLG could predict tumor stage. The ROC curve analysis of the SUVmean to predict ED-SCLC. With an SUVmean of 7.69 as the threshold, the sensitivity and specificity in the prediction of ED-SCLC were 78.72% and 47.22%, respectively. The AUC was 0.628 (95% CI: 0.535–0.715; *p =* 0.0166). The ROC curve analysis of the MTV to predict ED-SCLC. With an MTV of 61.36 as the threshold, the sensitivity and specificity in the prediction of ED-SCLC were 82.98% and 66.67%, respectively. The AUC was 0.823 (95% CI: 0.742–0.887; *p <* 0.0001). The ROC curve analysis of the TLG to predict ED-SCLC. With a TLG of 405.85 as the threshold, the sensitivity and specificity for the prediction of ED-SCLC were 80.85% and 63.89%, respectively. The AUC was 0.779 (95% CI: 0.694–0.850; *p <* 0.0001).

In patients with SCLC, SUVmean, MTV, and TLG were higher in ED-SCLC compared to those in LD-SCLC, respectively (*p* > 0.05), whereas SUVmax in patients with ED and those with LD were not significantly different (**Table 3**). With a cutoff value of 7.69, 61.36, and 405.85, high MTV and TLG could separately predict ED-SCLC (*p* < 0.05, **Figure 2**), while ROC analysis revealed that SUVmax could not predict ED-SCLC (*p* = 0.123 and 0.087). There were no significant differences in SUVmax, SUVmean, MTV, and TLG observed in SCLC patients with different age and smoking (**Table 3**).

**Table 3:** Analysis of metabolic parameters of SCLC on PET/CT scanning (*n* = 119).

	SUVmax	*p*	SUVmean	*p*	MTV (cm^3^)	*p*	TLG (g)	*p*
**Age**		0.987		0.782		0.564		0.644
≤64	12.83 (5.39, 47.34)		6.84 (3.33, 21.49)		71.28 (4.29, 1,208.91)		476.35 (28.85, 5,530.39)	
>64	12.78 (5.85, 25.53)		7.02 (3.17,20.88)		64.34 (2.95, 1,091.43)		461.58 (19.77, 6,965.86)	
**Gender**		0.817		0.332		0.040		0.096
Male	12.78 (5.39, 47.34)		6.86 (3.17, 21.49)		71.28 (2.95, 1,208.91)		482.41 (19.77, 6,965.86)	
Female	12.88 (7.06, 21.20)		7.42 (3.90,13.28)		31.92 (7.24, 120.97)		237.66 (34.54, 1,606.65)	
**Smoking**		0.172		0.133		0.211		0.198
Yes	13.02 (5.39, 47.34)		7.07 (3.17, 21.49)		72.13 (2.95, 1,208.91)		487.74 (19.77, 6,965.86)	
No	11.91 (6.12, 25.53)		6.24 (3.49, 11.94)		41.58 (7.24, 1,091.43)		317.70 (34.54, 5,556.47)	
**Tumor Stage**		0.788		0.018		0.000		0.000
LD-SCLC	12.78 (5.85, 26.12)		7.42 (3.32, 14.88)		38.93 (2.95, 299.21)		290.37 (19.77, 4,267.74)	
ED-SCLC	12.83 (5.39, 47.34)		6.43 (3.17, 36.25)		161.81 (7.58, 1208.91)		1126.64 (43.66, 6,965.86)	

#### Correlation of high metabolic parameters of SCLC with increased inflammatory markers

All SCLC patients were divided into low-MTV (or low-TLG) and high-MTV (or high-TLG) groups by cutoff values of 61.36 (or 405.85) (the cutoff value predicting ED-SCLC). The results showed that the NLR, PLR, MLR, and SII were higher in the high-MTV or high-TLG patients than in the low-MTV or low-TLG patients, respectively (*p* < 0.05, **Table 4**).

**Table 4:** Correlation of inflammatory markers with different MTV or TLG levels of SCLC.

	MTV (cm^3^)		*p*	TLG (g)		*p*
	≤61.36 (56)	>61.36 (63)		≤405.85 (55)	>405.85 (64)	
NLR	2.13 (0.30, 6.31)	3.13 (1.00, 17.86)	0.000	2.14 (0.30, 8.69)	3.12 (1.00, 17.86)	0.001
PLR	118.22 (48.70, 384.62)	164.71 (154.40, 561.19)	0.001	120.30 (48.70, 384.62)	164.30 (54.40, 561.19)	0.001
MLR	0.25 (0.08, 0.81)	0.34 (0.04, 2.49)	0.008	0.26 (0.08, 0.81)	0.34 (0.04, 2.49)	0.019
SII	438.80 (47.88, 2233.15)	750.17 (136.54, 2410.20)	0.000	443.40 (47.88, 2233.15)	743.82 (136.54, 2410.20)	0.000

#### Correlation of co-high metabolic parameters/inflammatory markers with binary stage of SCLC

Patients with SCLC were grouped into ^co-low^ MTV/NLR or ^co-low^ MTV/MLR or ^co-low^ MTV/SII, ^low^ MTV/^high^ NLR or ^low^ MTV/^high^ MLR or ^low^ MTV/^high^ SII, ^high^ MTV/^low^ NLR or ^high^ SUVmax/^low^ MLR, and ^co-high^ MTV/NLR or ^co-high^ MTV/MLR groups, respectively, based on the corresponding cutoff values (the cutoff value predicting ED-SCLC). TLG was the same as above. The results showed that the incidences of ^Co-high^ MTV/NLR, ^Co-high^ MTV/PLR, ^Co-high^ MTV/MLR, ^Co-high^ MTV/SII, ^Co-high^ TLG/NLR, ^Co-high^ TLG/PLR, ^Co-high^ TLG/MLR, and ^Co-high^ TLG/SII were higher in ED-SCLC patients than those in LD-SCLC, respectively (*p* = 0.000, **Table 5**). The incidences of ^Co-high^ MTV/NLR, ^Co-high^ MTV/MLR, ^Co-high^ MTV/SII, ^Co-high^ TLG/NLR, ^Co-high^ TLG/MLR, and ^Co-high^ TLG/SII were higher in male patients than those in female patients, respectively (**Table 5**). The incidences of ^Co-high^ MTV/MLR and ^Co-high^ TLG/MLR were higher in patients older than 64 years. However, the MTV and TLG of all lesions and NLR, PLR, MLR, or SII status did not exhibit a significant relationship with smoking.

**Table 5:** Relationship of metabolic parameters and inflammatory markers with binary stage of SCLC.

	Tumor Stage			Gender		
	LD-SCLC	ED-SCLC	*p*	Female	Male	*p*
^Co-low^ MTV/NLR	37	2	0.000	9	30	0.013
^Low^ MTV/^High^ NLR	11	6		0	17	
^High^ MTV/^Low^ NLR	10	11		3	18	
^Co-high^ MTV/NLR	14	28		2	40	
^Co-low^ MTV/PLR	44	6	0.000	9	41	0.207
^Low^ MTV/^High^ PLR	4	2		0	6	
^High^ MTV/^Low^ PLR	14	20		2	32	
^Co-high^ MTV/PLR	10	19		3	26	
^Co-low^ MTV/MLR	36	3	0.000	9	30	0.007
^Low^ MTV/^High^ MLR	12	5		0	17	
^High^ MTV/^Low^ MLR	15	13		4	24	
^Co-high^ MTV/MLR	9	26		1	34	
^Co-low^ MTV/SII	39	4	0.000	9	34	0.035
^Low^ MTV/^High^ SII	9	4		0	13	
^High^ MTV/^Low^ SII	9	13		3	19	
^Co-high^ MTV/SII	15	26		2	39	
^Co-low^ TLG/NLR	36	2	0.000	9	29	0.012
^Low^ TLG/^High^ NLR	10	7		0	17	
^High^ TLG/^Low^ NLR	11	11		3	19	
^Co-high^ TLG/NLR	15	27		2	40	
^Co-low^ TLG/PLR	43	6	0.000	9	40	0.183
^Low^ TLG/^High^ PLR	3	3		0	6	
^High^ TLG/^Low^ PLR	15	20		2	33	
^Co-high^ TLG/PLR	11	18		3	26	
^Co-low^ TLG/MLR	35	3	0.000	9	29	0.007
^Low^ TLG/^High^ MLR	11	6		0	17	
^High^ TLG/^Low^ MLR	16	13		4	25	
^Co-high^ TLG/MLR	10	25		1	34	
^Co-low^ TLG/SII	38	4	0.000	9	33	0.033
^Low^ TLG/^High^ SII	8	5		0	13	
^High^ TLG/^Low^ SII	10	13		3	20	
^Co-high^ TLG/SII	16	25		2	39	

Univariate analysis revealed that ^Co-high^ MTV/NLR (*p* = 0.000), ^Co-high^ MTV/NLR (*p* = 0.001), ^Co-high^ MTV/MLR (*p* = 0.000), ^Co-high^ MTV/SII (*p* = 0.000), ^Co-high^ TLG/NLR (*p* = 0.000), ^Co-high^ TLG/PLR (*p* = 0.005), ^Co-high^ TLG/MLR (*p* = 0.000), ^Co-high^ TLG/SII (*p* = 0.001), and smoking were related to the binary stage of SCLC (**Table 6**). Multivariate analysis further revealed that only ^Co-high^ MTV/MLR [odds ratio (OR): 8.67, 95% CI: 3.51–21.42, *p* = 0.000] was an independent predictor for ED-SCLC (**Table 6**). However, the gender and age did not exhibit a significant relationship with the binary stage of SCLC (**Table 6**).

**Table 6:** Univariate and multivariate logistic regression analysis of potential relationships between patients’ characteristics and binary stage of SCLC.

	Univariate*p*-value	Multivariate*p-*value	OR	95% CI for OR	
				Lower	Upper
Gender	0.057	0.184	4.50	0.96	21.12
Age	0.910	0.314	1.04	0.50	2.12
Smoking	0.042	0.163	2.81	1.04	7.62
^Co-high^ MTV/NLR	0.000	0.241	6.11	2.68	13.93
^Co-high^ MTV/PLR	0.001	0.416	4.21	1.73	10.21
^Co-high^ MTV/MLR	0.000	0.000	8.67	3.51	21.42
^Co-high^ MTV/SII	0.000	0.270	4.71	2.10	10.56
^Co-high^ TLG/NLR	0.000	0.437	5.13	2.28	11.54
^Co-high^ TLG/PLR	0.005	0.615	3.44	1.44	8.22
^Co-high^ TLG/MLR	0.000	0.405	7.05	2.92	16.99
^Co-high^ TLG/SII	0.001	0.432	3.98	1.79	8.84

## Discussion

In this study, our results revealed that the baseline inflammatory markers (NLR, MLR, PLR, and SII) and metabolic parameters (MTV and TLG) were significantly correlated with the binary stage of SCLC. In addition, hematological parameters (NLR, MLR, PLR, and SII) were significantly associated with MTV and TLG in SCLC patients. More importantly, co-high semi-quantitative parameters (MTV and TLG) and hematological parameters (NLR, MLR, PLR, and SII) were significantly related to ED-SCLC, but only ^Co-high^ MTV/MLR was identified as an independent predictor for ED-SCLC.

Growing evidence has demonstrated that inflammatory markers (NLR, PLR, MLR, and SII) in peripheral blood have been suggested to be correlated with the stage of different tumors, such as NSCLC (10), renal cell carcinoma (11), and colon cancer (12). Oner et al. (11) demonstrated that NLR and LMR predicted the late stage in renal cell carcinoma. In a study of colon cancer and NSCLC, Uludag et al. (12) and Goksel et al. (10) showed that NLR and PLR were significantly higher in late stage than those in early stage. In accordance with the previous studies, our study suggested that hematological parameters (NLR, PLR, MLR, and SII) were correlated with the binary stage in patients with SCLC. High NLR, MLR, and SII can be caused by increased neutrophils and monocytes or/and decreased lymphocytes in peripheral blood. Inflammation cells are known to be considered as part of the tumor microenvironment and promote development (14). Monocytes or neutrophils could directly form complexes with tumor cells and mediate migration in blood vessel (15). The complexes help metastatic seeds escape immune surveillance, while lymphocytes prevent the development of cancer by secreting protective inflammatory factors (16). Platelets also secrete inflammatory factors (e.g., vascular endothelial growth factor, VEGF) to facilitate tumor angiogenesis and metastasis (17). All the above theories suggested that baseline NLR, MLR, PLR, and SII might predict ED SCLC.

SUVmax reflects the maximum value of tumor metabolism. However, MTV and TLG are calculated based on the volume of interest (VOI); thus, they may be better to reflect tumor metabolism and burden. In the previous study, ^18^F-FDG PET/CT is used as a reliable molecular imaging method for staging patients with SCLC (5). In the present study, we also explored the application of semi-quantitative parameters *via* ^18^F-FDG PET/CT to assess the binary stage in patients with SCLC. Our study demonstrated that SUVmean, MTV, and TLG were related to the binary stage of SCLC patients, but SUVmax was not. Apostolova et al. (18) reported that MTV and SUVmax were associated with stage in patients with NSCLC. In another study, Dolan et al. (19) demonstrated that an elevated TLG was correlated with TNM stage of NSCLC. In addition, Hu et al. (20) found that MTV and TLG were related to the stage in patients with adenocarcinoma, and only MTV was associated with stage in patients with squamous cell carcinoma. Based on the above findings, the relationships between different metabolic parameters and the stage of lung cancer were not identical, but they could be used to evaluate the tumor stage for lung cancer. Although the pathological type and tumor stage in the present study are different from the previous studies, our results also supported this.

In addition, with the increase of MTV and TLG of SCLC patients, hematological parameters of NLR, PLR, MLR, and SII were elevated. A previous study reported that there were positive correlations between NLR and metabolic parameters (SUVmax, SUVmean, MTV, TLG, whole-body MTV, and TLG) *via* PET/CT in patients with SCLC (21). However, the PLR, MLR, and SII were respectively associated with MTV and TLG of SCLC in our study, which have not been reported before. To our knowledge, the underlying mechanism of relationship between inflammatory markers and metabolic parameters is undergoing investigation. A similar relationship between metabolic parameters and inflammatory markers was demonstrated in other cancers including colorectal cancer (22) and NSCLC (10). Xu et al. (22) suggested that SUVmax, MTV, and TLG were significantly associated with LMR and NLR. In a study of NSCLC, Goksel et al. (10) reported that MTV and TLG were positively related to NLR and PLR. These relationships between semi-parameters and hematological parameters in patients with different malignancies may be explained by certain opinions. On the one hand, inflammatory cells infiltrate primary tumors, resulting in the increase of ^18^F-FDG uptake (23). On the other hand, the hypoxia promotes the secretion of VEGF by inflammatory cells, resulting in tumor angiogenesis and increase of ^18^F-FDG uptake within tumor (24). The local tumor metabolism may have resulted from tumor metabolic itself and inflammatory cells (25). Interestingly, in the present study, we observed that the co-high MTV (or TLG) and inflammatory markers (NLR, PLR, MLR, and SII) were associated with ED-SCLC, but only ^co-high^ MTV/MLR was considered as an independent predictor for ED-SCLC. MTV represents the metabolic tumor volume of all lesions, and MLR reflects the host’s systemic inflammatory response. These findings mean that ^co-high^ MTV/MLR might be not only more accurate, but also effective for detecting ED-SCLC in the present study. Therefore, the correlation between metabolic parameters *via* ^18^F-FDG PET/CT and inflammatory markers needs further research. In a word, our results preliminarily demonstrated the synergistic effect of tumor metabolic activity with inflammatory markers in assessing the binary stage of SCLC.

SCLC has a close association with smoking, which is considered a factor in the development of SCLC (26, 27). Smoking has been proven to associate with inflammatory markers such as NLR, eosinophil-to-lymphocyte ratio (ELR), and lymphocyte-to-monocyte ratio (LMR) (28). In this study, although there was no significant correlation between inflammatory markers of NLR, PLR, MLR, and SII or metabolic parameters with smoking status, the incidences of ^Co-high^ MTV/MLR, ^Co-high^ MTV/SII, ^Co-high^ TLG/MLR, and ^Co-high^ TLG/SII were higher in smokers than nonsmokers. Furthermore, smoking was not an independent predictor for the binary stage of SCLC, which is due to only few patients being never-smokers in this study. However, understanding the association between smoking, inflammation markers, tumor metabolism, and binary stage in SCLC patients needs additional research in the future.

There are some limitations in this study. Firstly, as a retrospective study, there are some limitations such as a great gender ratio difference, the lack of a control matched group, and the absence of a rigorous control of the inflammation-related lung diseases (e.g., obstructive pneumonia, interstitial pneumonia, and chronic obstructive pulmonary disease). Secondly, the sample size was relatively small and all patients were only from a single center. A multi-center prospective study with a larger sample size should be carried out in the future.

## Conclusion

In conclusion, our results demonstrate that the baseline inflammatory markers (NLR, MLR, and SII) and tumor metabolic parameters are associated with the binary stage in patients with SCLC. Moreover, the co-high MTV/MLR based on metabolic tumor volume and systemic inflammatory response could be of help for predicting the ED-SCLC. However, further investigation needs to evaluate the combined role of inflammatory markers and tumor metabolic parameters *via* PET/CT in detecting ED-SCLC at baseline.

## Data availability statement

The original contributions presented in the study are included in the article/Supplementary Material. Further inquiries can be directed to the corresponding author.

## Ethics statement

The studies involving human participants were reviewed and approved by the Ethics Committee of the First Affiliated Hospital of Nanjing Medical University. The patients/participants provided their written informed consent to participate in this study.

## Author contributions

JS, YH, YL, DL, TL, and YXH directly participated in the planning, execution or analysis of the study. JS, YH, and TL designed the study and interpreted the results. DL and YL contributed to collection of clinical dates. JS and YH drafted the manuscript. All authors contributed to the article and approved the submitted version.

## Funding

This work was supported by grants from the National Jiangsu Provincial Health and Family Planning Commission Foundation (H2018029) and Research Project of Jiangsu Cancer Hospital (No. ZJ202122). These funders did participate in design, data collection, and analysis of the present study and have no role in the decision to publish the manuscript.

## Conflict of interest

The authors declare that the research was conducted in the absence of any commercial or financial relationships that could be construed as a potential conflict of interest.

## Publisher’s note

All claims expressed in this article are solely those of the authors and do not necessarily represent those of their affiliated organizations, or those of the publisher, the editors and the reviewers. Any product that may be evaluated in this article, or claim that may be made by its manufacturer, is not guaranteed or endorsed by the publisher.
